# Reporting dream experience: Why (not) to be skeptical about dream reports

**DOI:** 10.3389/fnhum.2013.00708

**Published:** 2013-11-07

**Authors:** Jennifer M. Windt

**Affiliations:** Department of Philosophy, Johannes Gutenberg-UniversityMainz, Germany

**Keywords:** dreaming, subjective experience, dream reports, first-person reports, philosophy of mind, scientific dream research, skepticism, inference to the best explanation

## Abstract

Are dreams subjective experiences during sleep? Is it *like* something to dream, or is it only like something to remember dreams after awakening? Specifically, can dream reports be trusted to reveal what it is like to dream, and should they count as evidence for saying that dreams are conscious experiences at all? The goal of this article is to investigate the relationship between dreaming, dream reporting and subjective experience during sleep. I discuss different variants of philosophical skepticism about dream reporting and argue that they all fail. Consequently, skeptical doubts about the trustworthiness of dream reports are misguided, and for systematic reasons. I suggest an alternative, anti-skeptical account of the trustworthiness of dream reports. On this view, dream reports, when gathered under ideal reporting conditions and according to the principle of temporal proximity, are trustworthy (or transparent) with respect to conscious experience during sleep. The *transparency assumption* has the status of a methodologically necessary default assumption and is theoretically justified because it provides the *best explanation* of dream reporting. At the same time, it inherits important insights from the discussed variants of skepticism about dream reporting, suggesting that the careful consideration of these skeptical arguments ultimately leads to a positive account of why and under which conditions dream reports can and should be trusted. In this way, moderate distrust can be fruitfully combined with anti-skepticism about dream reporting. Several perspectives for future dream research and for the comparative study of dreaming and waking experience are suggested.

## Introduction

There is a tension in contemporary philosophical writing on dreams. On the one hand, dreams are commonly regarded as conscious experiences occurring during sleep (Metzinger, 2003; McGinn, 2004; Revonsuo, 2006; Windt and Metzinger, 2007; Ichikawa, 2009; Sosa and Ichikawa, 2009). On the other hand, dream reports are taken to be notoriously untrustworthy. Dream recall is a fleeting and highly unstable phenomenon, and research has shown that a majority of dreams are forgotten^1^. Such worries are strengthened by different versions of philosophical skepticism about the trustworthiness of dream reports. The reliance of scientific dream research on dream reports is, on this view, an obstacle to be overcome, resulting in the somewhat uncomfortable position that dream reports, while crucial to the study of dreaming, are less trustworthy than reports of waking experience (Nir and Tononi, 2010, p. 89).

The goal of this article is to show that skepticism about dream reporting is misguided. I argue that the reliance on dream reports is not only methodologically necessary and a precondition for scientific dream research, but also theoretically justified. At the same time, a recurrent theme of this article is that philosophical skepticism about dream reporting can be used as a foil for constructing a positive account of dream reporting. Hence my ambiguous subtitle: Taking skeptical doubts about the trustworthiness of dream reports seriously leads to an anti-skeptical account of why dream reports should, in fact, be trusted. In reporting dreams, we are actually reporting dream experience.

In the first section (“The *Transparency Assumption* of Dream Reporting and the *Experiential and Reportability Assumptions* of Dreaming”), I analyze the background assumptions behind scientific dream research and argue that it is constrained, for methodological reasons, by trusting dream reports. In the following section (“Skepticism About Dream Reporting”), I discuss three prominent versions of skepticism about dream reporting and show that they all fail to shed doubt on the trustworthiness of dream reports. Because this failure is systematic, I conclude that skepticism about dream reporting is generally misguided. Then, (in the section “Why to Trust Dream Reports”), I use insights from my discussion of skepticism about dream reporting to defend an anti-skeptical account, arguing that inference to the best explanation provides theoretical justification for saying that dream reports are trustworthy^2^. Several consequences and perspectives for scientific dream research are discussed in the final section (“The Way Forward: Consequences for Scientific Dream Research”).

## The *transparency assumption* of dream reporting and the *experiential* and *reportability assumptions* of dreaming

The *experiential assumption* says that dreams are subjective experiences occurring in sleep in the sense that they are phenomenal states, or that it is *like* something (Nagel, 1974) to dream. It is implicit in Aristotle's treatise on dreams, arguably the first systematic theory of dreaming and its physiological sources (Aristotle, 350 B.C.E./1994–2009; Dreisbach, 2000; Barbera, 2008) and lies at the root of the epistemological problem of whether dreams can be distinguished from wakefulness and whether we can ever rule out, at any given moment, that we are now dreaming (Descartes, 1641/1901). It is also commonly assumed by contemporary theories of dreaming, both from philosophy of mind and cognitive neuroscience (cf. Windt, in press).

**Figure 1:**
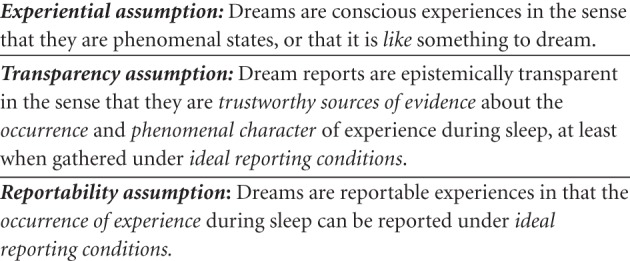
**The methodological background assumptions behind scientific dream research**.

By contrast, the trustworthiness of dream reports only became a target of philosophical discussion in the second half of the 20th century. This shift in the philosophical debate from dream experience to dream reporting was not theoretically neutral: skepticism about dream reporting was used to defend skepticism about dream experience (see the section “Skepticism About Dream Reporting” for details). Importantly, this shift revealed the deep connection between the *experiential* and *transparency assumptions*: Because access to dream experience is always indirect via retrospective dream reports, claims about dream experience, at least implicitly, assume that dream reports are trustworthy sources of evidence about subjective experience during sleep^3^. Consequently, if the *transparency assumption* is rejected, the grounds for endorsing the *experiential assumption* are eroded: if dream reports are not trustworthy with respect to dream experience, then there is no reason to claim that dreams are experiences in the first place. Indeed, the *experiential assumption*, on this view, is a simplified and incomplete formulation of the *transparency assumption* (see Figure 1).

A brief look at the beginnings of scientific dream research illustrates this point. In 1953, Eugene Aserinsky and Nathaniel Kleitman published a seminal article describing the discovery of regularly occurring periods of eye motility during sleep and their correlation with dream reports after awakening. Describing the results of their study, Aserinsky and Kleitman noted that “of 27 interrogations during [sic] ocular motility, *20 revealed detailed dreams* usually involving visual imagery” (Aserinsky and Kleitman, 1953, p. 273; my emphasis) and suggested that the method of timed awakenings from rapid eye movement (short: REM) sleep “furnishes the means of determining the *incidence and duration of periods of dreaming*” (Aserinsky and Kleitman, 1953, p. 274; my emphasis). Similarly, in an article titled *The relation of eye movements during sleep to dream activity: An objective method for the study of dreaming*, William Dement and Nathaniel Kleitman stated that “the results of these experiments indicate that *dreaming* accompanied by REM's and a low-voltage electroencephalogram occurred periodically in discrete episodes during the course of a night's sleep” (Dement and Kleitman, 1957; p. 345; my emphasis).

It is noteworthy that the authors of both articles used the correlation between eye movements during sleep and retrospective dream reports to infer a direct association between the physiological characteristics of REM sleep and dreaming. Likewise, subjects' frequent inability to *report* dreams following awakenings from periods of sleep lacking REMs (or NREM sleep) was interpreted as strong evidence for the absence of *dreaming* during NREM sleep. This is not to say that dream reports were regarded as stand-alone evidence of previous dreaming; clearly, the significant association with eye movements and EEG measures of high cortical activity supported their reasoning. Yet, it is equally clear that reports of dreaming and of nondreaming were afforded equal *prima facie* credibility and that this assumption was crucial for establishing the association between dreaming and REM sleep, as well as the absence of dreaming during NREM sleep, in the first place^4^. These early researchers very naturally took the association between dream reporting and dreaming to be such that reports after awakening licensed direct inferences about dreaming (or its absence) in the preceding sleep stage. Consequently, we can now say that the *transparency assumption* extends to reports of nondreaming as well, thus giving rise to the further assumption that dreams, at least under ideal conditions (e.g., following timed awakenings in the sleep laboratory), are *reportable* experiences during sleep (see Figure 1; cf. Windt, in press). Indeed, this suggests that the endorsement of all three assumptions is necessary for saying that *dream* research, as opposed to research on dream reports, is possible.

An obvious objection would be that this formulation is too permissive: subjects often say that they have forgotten parts of their dream and they may even have the impression of having had a vivid dream without being able to remember any details at all. In one study, more than a third of reports from NREM sleep described such white dreams (Noreika et al., 2009). This suggests that the *reportability assumption* should not be understood as demanding that all details of an experience can be reported upon awakening: after all, one can report the *occurrence* of a detailed experience without being able to give an equally detailed report. Reports are descriptions, and they will necessarily be incomplete and selective. Yet, assuming that dreams are at least in principle reportable experiences (or that they could be reported under ideal reporting conditions, for instance following timed awakenings in the sleep laboratory) is an important condition of possibility for scientific dream research: without it, dream experience becomes decoupled from the primary sort of evidence used for its investigation.

## Skepticism about dream reporting

The claim that *scientific* dream research is constrained by the space of reportable dreams under the assumption of transparency may sound preposterous. While there is widespread agreement that scientific *consciousness* research requires the systematic investigation of subjective experience, the development of valid first-person methods is often considered as one of its main challenges (Dennett, 1991; Varela, 1996; Thompson, 2007; Overgaard et al., 2008; Froese et al., 2011). If first-person reports turned out to be systematically unreliable (Schwitzgebel, 2011), or if phenomenal experience turned out to be too elusive to be cognitively accessible and hence reportable, this would threaten the possibility of scientific consciousness research altogether (Block, 2007, 2011; Cohen and Dennett, 2011).

In this climate, the prospects for scientific dream research may seem particularly bleak. While recent decades have seen a sharp increase in publications on consciousness, the same has not been true for dreaming, and the fields of dream and consciousness research are largely separate (Wamsley, 2013; Windt, in press). Dream researchers themselves have long portrayed the reliance on dream reports as a weakness, suggesting that in order to make real progress, the trustworthiness of dream reports would have to be verified by independent means or that dream research should aim to move beyond dream reports altogether (e.g., Hall and Van de Castle, 1966; Winget and Kramer, 1979; Leclair-Visonneau et al., 2010). In philosophy, two main types of skepticism about dream reporting can be distinguished. The first accepts that there is a deep, perhaps even conceptual connection between dreaming and dream reporting, but argues that this very fact speaks against, rather than for, a naturalistically minded approach to dreaming and its occurrence during different sleep stages (Malcolm, 1956, 1959/1962; Squires, 1973, 1995; McFee, 1994; Schröder, 1997). The second regards the question of dream experience as an empirical question, but claims that dream reports are too untrustworthy to study dream experience in any detail (Dennett, 1976; Schwitzgebel, 2011; Rosen, 2013). Either way, the strategy of using dream reports for the scientific investigation of conscious experience during sleep is threatened. This section discusses three particularly prominent versions of skepticism about dream reporting and shows that they all, if successful, would have consequences beyond dreaming for the use of first-person reports in consciousness research.

### Norman malcolm's rejection of dream experience

In the late 1950s, and in direct reaction to the study by Dement and Kleitman discussed above, Norman Malcolm mounted his attack against the *experiential assumption* of dreaming by applying a particularly strict type of verificationism to the concept of dreaming. Following Wittgenstein (1953), Malcolm assumed that the meaning of a concept is wholly determined by the criteria used for its ascription. On this view, the concept of dreaming is special because it has no present-tense behavioral criterion: the sole criterion of dreaming is the retrospective report of having dreamt. “What we must say, although it seems paradoxical, is that the concept of dreaming is derived, not from dreaming, but from descriptions of dreams, i.e., from the familiar phenomenon that we call ‘telling a dream”’ (Malcolm, 1959/1962, p. 55; cf. Wittgenstein, 1953, p. 184). Consequently, there can be no ongoing behavioral evidence for saying that a person is now asleep and dreaming: any such behavior (such as sleeptalking) would show that the person was, in fact, partially awake. Though we use the same language to describe dreams and waking experiences, “it is fruitless to argue that this identity of language is due to an identity of experience that lies behind it” (Malcolm, 1959/1962, p. 94): “If a man had certain thoughts and feelings in a dream it no more follows that he had those thoughts and feelings while asleep, than it follows from his having climbed a mountain in a dream that he climbed a mountain while asleep” (Malcolm, 1959/1962, pp. 51–52). Descartes' (1641/1901) worry that we are deceived by our dreams is, according to Malcolm, incoherent: one cannot, while dreaming, mistakenly think that one is awake: “to a person who is sound asleep, ‘dead to the world,’ things cannot even seem” (Malcolm, 1956, p. 26).

Malcolm's rejection of the *experiential assumption* is based on purely conceptual considerations: “if a person is in *any* state of consciousness it logically follows that he is not sound asleep” (Malcolm, 1956, p. 21). While dream reports, as the sole criterion of dreaming, are transparent with respect to *dreams* by matter of logical necessity, this does not entail (and indeed precludes) their transparency with respect to subjective *experience* during sleep. Indeed, the very idea that dream reports literally reveal what was *experienced* during sleep is misconceived: “To find out one dreamt the incident is to find out that the impression one had on waking is false” (Malcolm, 1959/1962, p. 64).

Importantly, Malcolm's analysis of dreaming amounted to a wholesale rejection of the very possibility of scientific dream research. In particular, he accused Dement and Kleitman of replacing the retrospective criterion of dream reporting with a new, physiological criterion. By introducing REM sleep as a third-person observable, present-tense criterion for the occurrence and duration of dreams, they had not only deprived dream reports of their sole authority, but had changed the concept of dreaming itself. The very project of conducting scientific *dream* research beyond the mere study of dream reports was, to Malcolm, founded on a misconception of the nature of the concept of dreaming. In trying to investigate dreaming in the laboratory, these researchers had inadvertently changed the explanatory target from dreaming to something else.

Malcolm's analysis of dreaming engendered an enormous amount of criticism. Its early impact is reflected in Dunlop's^1997^ collection of *Philosophical Essays on Dreaming*, and to this day, most philosophical publications on dreaming discuss his views at least in passing. For present purposes, the most important point is that the debate about Malcolm's *Dreaming* can be described as a proxy war on more general issues. As several of Malcolm's critics pointed out, his views not only failed with respect to the concept of dreaming in particular, but because they assumed an overly strict brand of verificationism, an implausible account of meaning and justification, and misconstrued the nature of philosophical methodology.

The description of the debate on Malcolm's *Dreaming* as a proxy war is nicely reflected by Hilary Putnam: “Why is it important to show that Malcolm is wrong? Malcolm's is the sharpest statement of Verificationism in the 1950s. If Malcolm is right, then the ‘naïve’ way of understanding our language and our knowledge is wrong” (Putnam, 1986, p. 306). This is not the place to enter into a discussion on the relationship between meaning, linguistic intelligibility and justification. For present purposes, it is sufficient to note that Malcolm's argument depends on an implausibly strong form of logical behaviorism and that without it, his skeptical arguments about dreaming and dream reporting, as well as his reasons for denying the possibility of scientific dream research, evaporate. That this is indeed the case was an important result from the critical literature. For instance, Putnam notes that linguistic intelligibility does not depend on criteria with which the truth of a sentence can be verified with absolute certainty, but on its “ability to occur in coherent and appropriate discourses, on paraphrasability” (Putnam, 1986, p. 309). Similarly, Chihara and Fodor accuse Malcolm of misconstruing the nature of justification. Justification cannot be made contingent on criteria, but rather depends “on appeals to the simplicity, plausibility, and predictive adequacy of an explanatory system as a whole” (Chihara and Fodor, 1965/1977, p. 197). Consequently, the *experiential assumption* can no longer be ruled out as a matter of logical necessity and, contra Malcolm, it is not at all clear that psychological sentences lose their meaning when applied to dreaming. It is also no longer clear that the use of physiological evidence automatically changes the concept of dreaming. This is an important first step toward making genuinely scientific dream research possible.

In this context, note that the debate between Malcolm and his critics can also be viewed as a proxy war on philosophical methodology and the relationship between armchair conceptual analysis, first-person reports, and scientific research. Deprived of its basis in a convincing theory of meaning and justification, Malcolm's position is now revealed to result from a somewhat arbitrary form of conceptual legislation. In particular, Malcolm's argument presupposes a misguided view of analyticity, according to which philosophers conducting armchair conceptual analysis have privileged access to deep conceptual truths that are hidden to laypersons and researchers alike. As Putnam puts it, “the lexicographer would undoubtedly perceive the logical (or semantical) connection between being a pediatrician and being a doctor, but he would miss the *allegedly* ‘logical’ character of the connection between dreams and waking impressions. […] this ‘depth grammar’ kind of analyticity (or ‘logical dependence’) does not exist” (Putnam, 1986, p. 306).

The problem with Malcolm's conceptual analysis of dreaming and dream reporting, however, runs even deeper. Even granting, for the sake of argument, that dreams are *not* experiences for conceptual reasons—or even that there is a conceptual contradiction in saying that one had certain experiences during sleep—this has no direct consequences for questions concerning the phenomenology of dreaming. The *experiential assumption*, as introduced above, is not about the conceptual, but about the phenomenological analysis of dream experience, and a point that Malcolm consistently misses is that there is simply no necessary entailment from purely conceptual or analytic arguments to facts about phenomenal experience. From now on, let's call the practice of deriving claims about phenomenal experience from conceptual analysis alone the *fallacy from armchair conceptual analysis*, short the *armchair fallacy*^5^. Asking whether a dream occurred during a given period of sleep, or which types of imagery or emotions occurred in a given in dream, is to ask *factual* questions about *what it is like* to dream (or whether it *was like* anything to undergo the preceding period of sleep); dream reports, under the assumption of transparency, provide the primary type of evidence for answering them. While there is certainly room for conceptual questions regarding dream experience, such questions are, at least in principle, wholly separable from questions about what it was like to have the relevant experiences. As Nagel points out, “it is a mistake to invest the demonstration that it is impossible to have experiences while asleep with more import than it has. It is an observation about our use of the word ‘experience’, and no more. It does not imply that nothing goes on in our minds while we dream” (Nagel, 1959, p. 114). Put this way, it becomes clear that independently of whether or not we *say* that dreams are experiences, this has no consequences whatsoever for the question of whether or not dreams are phenomenal states. The *experiential assumption* only says that dreams are experiences in the sense of being phenomenal states, or that it is *like* something to dream.

On this view, it might seem that nothing much hangs on such purely conceptual decisions. Yet, to the extent that a conceptual framework—for instance for the description of dreams and their relation to waking consciousness—aims to capture actual phenomena, it should strive toward empirical plausibility. If this is correct, then conceptual analysis not only fails to rule out the possibility of scientific dream research—quite to the contrary, it should be informed by empirical research results. Ideally, a philosophical framework should help structure the debate on dreaming and thus facilitate an integrated and interdisciplinary approach to dreaming and waking consciousness. At the very least, it should not hinder this process.

My preliminary conclusion is that conceptually motivated skepticism about dreaming and dream reporting, of which Malcolm's argument is a paradigmatic example, fails, and that it does so for principled reasons. Yet, this failure is informative: it shows that questions about dreaming and dream reporting cannot be construed as purely conceptual questions, and it suggests a more constructive way of describing the relationship between philosophical analysis and scientific research.

### Daniel C. Dennett's Cassette theory of dreaming

Perhaps the second most influential publication on dreams following Malcolm's *Dreaming* was Dennett's (1976) article *Are Dreams Experiences?*. Here, Dennett used an extended thought experiment to propose an alternative to the received view of dreaming. Importantly, his aim was not so much to answer the question of whether dreams are experiences, but rather “to treat the question itself as the specimen to be examined” (Dennett, 1979, p. 317). His main point was that this question cannot be settled by armchair conceptual analysis or on the basis of subjective testimony, but “only by the triumph of a good empirical theory over rival empirical theories” (Dennett, 1979, p. 317).

Dennett (1976) begins by construing a maximally strong case for scientific dream research. Having established the physiological correlates of dreaming, such as REM periods and wake-like EEG activity, a neurophysiological model of dreaming could make all sorts of predictions about the occurrence, timing, and duration of dreams and perhaps even translate physiological measures, such as REMs, into dream narratives. A sophisticated theory of dreaming might eventually posit the existence of three separate processes: a presentation process, which would be the physiological correlate of the ongoing dream, a memory-loading process, responsible for the subject's ability to recall the dream upon awakening, and a composition process, responsible for the composition of dream narratives. Once they had been identified, these processes could be tampered with—possibly to the point of obliterating veridical dream memories and substituting them with undreamed but nonetheless recalled dream narratives (Dennett, 1976, p. 156).

Next, Dennett suggests a more parsimonious view requiring only two of these processes. On the cassette theory of dreaming, an unconscious composition process during REM sleep prepares ‘dream cassettes’ for instantaneous insertion into memory upon awakening. While the cassette theory could accommodate all available physiological data on REM sleep, as well as the occasional incorporation of external stimuli into dreams and the apparent content-relativity of REMs (the so-called “scanning hypothesis”), it is radically different from the received view of dreaming in an important respect: “on the cassette theory it is not like anything to dream, although it is like something *to have dreamed*. On the cassette theory, dreams are not experiences we have during sleep; where we had thought there were dreams, there is only an unconscious composition process and an equally unconscious memory-loading process” (Dennett, 1976, p. 161). Dennett's key claim is that everyday experience could provide no evidence for or against this theory: *ex hypothesi*, the subject reporting a dream would be incapable of distinguishing real and instantaneously inserted dream memories.

On its face, Dennett's analysis of dreaming is less radical than Malcolm's in that it does not outright deny the *experiential assumption*. Because Dennett insists that the experiential character of dreaming is an open question, the *experiential assumption* might even turn out to be correct. On second sight, however, Dennett's view is at least as damaging to the *transparency assumption* of dream reporting as Malcolm's, because it denies that dream reports have any evidential status with respect to previous dream experience whatsoever. Note that even if the *experiential assumption* turned out to be correct, dream reports would not, indeed *could* not play any evidential role in showing this to be the case. Dennett (1976, p. 159) points out that he chose the cassette theory precisely because of its counterintuitivity: if we cannot even rule out such a radically different theory of dreaming based on subjective dream recall, then dream recall is wholly irrelevant to the question of dream experience. Seen in this context, Dennett's argument is not so much a version of skepticism about dreaming as about dream reporting. Unlike Malcolmian skepticism, Dennett regards the question of dream experience as a wholly empirical matter, leading to the demand for a science of dreaming that solves the question of dream experience without ascribing any evidential role to dream reports. On this view, dreaming is not only wholly separable from dream recall on the level of conceptual analysis, but the scientific study of dreaming is also, at least potentially, *methodologically* independent of the study of dream reports. Dennett even states under which conditions the *experiential assumption* might be confirmed: “If it turns out that sleep, or at least that portion of sleep during which dreaming occurs, is a state of more or less peripheral paralysis or inactivity; if it turns out that most of the functional areas that are critical to the governance of our wide awake activity are in operation, then there will be good reason for drawing the lines around experience so that dreams are included. If not, there will be good reason to deny that dreams are experiences” (Dennett, 1976, p. 169).

At this point—and indeed this is the way Dennett's challenge to the received view of dreaming is sometimes portrayed—one might argue that the cassette theory has turned out to be empirically invalid (Revonsuo, 2006, p. 77). Three lines of evidence are relevant. First, imaging studies have shown that REM sleep is characterized by a shift in regional activation patterns compared to wakefulness and/or NREM sleep (Dang-Vu et al., 2007; Desseilles et al., 2011). Specifically, the pons, thalamus, temporo-occipital, motor, as well as the limbic and paralimbic areas (including the amygdala) are highly activated during REM sleep, while the dorsolateral prefrontal and inferior parietal cortices are comparatively quiescent. This activation pattern fits in nicely with the predominance of visual and motor imagery during dreams, the frequency of intense, often negative emotions and cognitive deficits such as the loss of self-awareness, mnemonic deficits and the delusional belief in the reality of dream events. According to Allan Hobson's prominent model of dreaming, brain-stem driven internal signal generation, combined with muscular atonia or REM sleep paralysis, contributes to the sensory input/motor output blockade, explaining how rich internal experience combines with behavioral passivity and lack of responsiveness to external stimuli (Hobson et al., 2000).

A second line of evidence comes from lucid dreams. Experienced lucid dreamers can not only become aware of the fact that they are dreaming during the dream state, but also often have some level of dream control (LaBerge et al., 1981; LaBerge, 2007). Importantly, they are able to control the direction of their eye movements during their lucid dreams, and because these dream-eye movements correspond to real-eye movements and are clearly identifiable on the EOG, lucid dreamers can use previously determined eye movement patterns to communicate to researchers, in real-time, that they are *now* lucid and engaging in certain lucid dream experiments. This suggests that certain kinds of behavioral reports are possible even during the dream state. Note, however, that in order to rule out false positives, retrospective confirmation is required (Erlacher et al., 2003; Dresler et al., 2011, 2012).

A third line of evidence comes from dream-enactment behavior (Nielsen et al., 2009), most prominently seen in patients with REM sleep behavior disorder (RBD; Schenck and Mahowald, 1996; Schenck, 2005; Leclair-Visonneau et al., 2010). These patients show complex behaviors such as running or fighting off an attacker during REM sleep, and they often report dreams involving the same actions after awakening. Due to the loss of muscular atonia during REM sleep, they appear to be literally acting out their dreams.

Taken together, all of this suggests that there is now physiological and behavioral evidence supporting the occurrence of dreams during sleep and thus speaking against the cassette theory of dreaming. Or does it? Note that Dennett's thought experiment is constructed in such a way that the cassette theory is supposed to be able to accommodate *all* of the findings of future dream research. For the sake of argument, let us consider how the determined skeptic might respond to these three putative arguments for the *experiential assumption*. On the cassette theory of dreaming, findings on regional shifts in brain activation patterns from imaging studies would be attributed to an unconscious composition process during sleep. Nothing in these imaging data, or so the proponent of the cassette theory might argue, requires a conscious presentation process during sleep. Two types of objections might be raised against evidence from lucid dreaming. One, lucidity could be explained by saying that the unconscious composition process inserted “traces of itself into the recording via the literary conceit of a dream within a dream” (Dennett, 1976, p. 161), thus resulting in the impression, after awakening, of having been able to “tinker” with or control the ongoing dream. Eye movement signals might be explained in the same manner. Two, and perhaps more importantly, the skeptic might grant that lucid dreams are experiences, but deny that findings from lucid dreaming can be generalized to nonlucid REM dreams. Indeed, one recent study suggested that lucid dreams do not, as previously assumed, occur in unequivocal REM sleep (LaBerge, 1990), but rather in a hybrid state between dreaming and wakefulness (Voss et al., 2009), so caution about generalizations seems warranted. Finally, similar concerns could be voiced about the example of dream-enactment behavior. Again, the skeptic could either propose that this behavior was caused by or perhaps even left traces in the unconscious composition process, thus explaining why the retrospective dream report described exactly the same behavior, or he might grant that dream-enactment behavior is related to real dream experience, whilst denying that the same is true for ordinary dreams in healthy subjects. Perhaps, only lucid dreams, or only the dream-enactment dreams of RBD patients, are conscious experiences during sleep.

Note that my purpose in strengthening the case of the Dennettian skeptic about dream reporting is not to suggest that this position is, in fact, correct. My point here is more subtle. In particular, I want to suggest that once the skeptical challenge to the *transparency assumption* arising from the cassette theory has been accepted—that is, once it has been accepted that the question of dream experience is an empirical question that has to be resolved without granting evidential status to dream reports—there is no conclusive way to rule out the cassette theory^6^. Hence, contrary to Dennett's position in *Are Dreams Experiences?*, the question of dream experience is not a genuine empirical question.

This reading is also compatible with Dennett's use of a thought experiment that is highly similar to the cassette theory in *Consciousness Explained*. Here, he raises the question of whether we are able to distinguish between genuine and hallucinated memories (Dennett, 1991, pp. 115–126). Referring to Orwell's (2012) 1984-scenario of history being rewritten by the *Ministry of Truth*, Dennett identifies “Orwellian revision” with post-experiential memory revision and contrasts it with the “Stalinesque method” of staging show trials based on false confessions and fictive evidence. While Orwellian revision and Stalinesque show trials both aim at the production of misleading archives, there seems to be a clear–cut difference between the retrospective fabrication of historical accounts and the actual staging of simulated events. According to Dennett, it is, however, an illusion to believe that this distinction between pre- and post-experiential memory production can be applied “all the way in” and on a very small time scale. “Here the distinction between perceptual revisions and memory revisions that works so crisply at other scales is no longer guaranteed to make sense. We have moved into the foggy area in which the subject's own point of view is spatially and temporally smeared, and the question *Orwellian or Stalinesque?* loses its force” (Dennett, 1991, p. 119). As is the case for dreaming, the subjective indistinguishability of these two possibilities leads Dennett to identify conscious experience with the production of memory traces: “The Multiple Drafts Model makes “writing it down” in memory criterial for consciousness […]. There is no reality of conscious experience independent of the effects of various vehicles of content on subsequent action (and hence, of course, on memory)” (Dennett, 1991, p. 132). On this view, conscious experience is probe-dependent; it cannot be conceived of independently of reporting (cf. Dennett and Akins, 2008). It is interesting to note that on this level, Dennett endorses something similar to the *transparency assumption*. However, he does so only with respect to a drastically altered conception of experience, according to which experience is made contingent upon retrospective report, rather than conceived of as separate from it.

The application of the same kind of thought experiment to reports of waking experience suggests that for Dennett, the problems raised by dreaming and dream reporting are no different from those presented by waking experience. Dreams are particularly vulnerable to skeptical objections because we can only report them retrospectively after awakening—but they are not uniquely vulnerable (cf. Dennett, 1976, p. 166). Once again, the philosophical debate on dreaming appears to be a proxy war for broader questions concerning the relationship between conscious experience, memory, and first-person reports.

My preliminary conclusion is that the attempt to construct the cassette theory as an *empirically* valid alternative to the *experiential assumption* fails: doing so leads to an insoluble form of skepticism. Yet, this seems to leave the received view of dreaming in an equally vulnerable position, because it suggests that no straightforward empirical defense of dream experience and dream reporting is forthcoming. Consequently, the finding that empirical evidence cannot distinguish between skepticism and anti-skepticism about dream reporting appears to be equally damaging to both views.

### Eric Schwitzgebels on dream color

In a number of recent publications, Schwitzgebel has defended “blanket skepticism” (Hurlburt and Schwitzgebel, 2007, p. 234) about first-person reports. He repeatedly contrasts his view with Cartesian dream skepticism, claiming to raise doubts about current conscious experience as “the last refuge of the skeptic against uncertainty” (Schwitzgebel, 2011, p. 117): “There are major lacunae in our self-knowledge that are not easily filled in, and we make *gross, enduring mistakes* about even *the most basic features of our currently ongoing conscious experience*, even in *favorable circumstances of careful reflection*, with remarkable regularity” (Schwitzgebel, 2011, pp. 118–119; my emphasis).

For present purposes, his argument is interesting not only because he is one of the few philosophers to explicitly defend a strong version of skepticism about first-person reports, but also because, like Dennett, he takes dreaming to be a particularly clear example of their untrustworthiness. Schwitzgebel's skepticism about dream reporting is, however, more limited in scope because it is restricted to the question of whether we dream in color or in black and white. Based on a review of historical studies on color in dreams, he found evidence for “an arc of opinion: before scientific psychology, a consensus or assumption that dreams are colored; divided opinions into the early twentieth century; a consensus that dreams typically have little color from about 1930 to 1960; and then a sudden overturning of that consensus in the 1960s” (Schwitzgebel, 2011, p. 5; cf. Schwitzgebel, 2002).

Schwitzgebel suggests three possible interpretations of these findings. The first is that dreams, influenced by the rise first of black-and-white and then color television, changed from colored to black and white and back to colored. Second, media exposure may have influenced only people's reports of dreaming in color while leaving their dreams themselves unchanged. Third, dreams might be indeterminate with respect to color, i.e., they might be neither black and white nor colored. Again, the change from black-and-white to color media may have influenced only the way dreams are reported.

Note that these three different interpretations amount to different skeptical scenarios regarding judgments about colored dreaming. The strongest skeptical challenge arises from the third possibility: if dreams are neither black and white nor colored, then reports of colored or black and white dreaming are generally mistaken. By contrast, if the second possibility turned out to be true, only one group of people—either those reporting colored dreams or those reporting black and white ones—is mistaken. And on the first interpretation, changes in reports of colored dreaming would not warrant any doubts about the trustworthiness of dream reporting.

Schwitzgebel repeatedly (2002; 2011; Schwitzgebel et al., 2006) describes the possibility of a change in dreaming as opposed to a change in dream reporting as unlikely, defending the claim that either all or a certain group of subjects have mistaken opinions about the occurrence of color in their dreams. The deeper point, however, is that because all three interpretations are possible, and given that none of them can be ruled out at the outset, this finding itself undermines, or so it might seem, the *transparency assumption* with respect to reports of colored dreaming. Schwitzgebel recommends agnosticism about colored dreaming: “I don't know, and you probably don't know, whether we dream in color or not. Although I have found in conversation that most people answer confidently when asked about the coloration or non-coloration of their dreams, that confidence is misplaced” (Schwitzgebel, 2011, pp. 3f).

Schwitzgebel's skepticism derives its force from the fact that it is neither based on conceptual considerations nor on a hypothetical thought experiment, but on actual empirical findings. His position is also special in that it has prompted a number of follow-up studies, including his own (Schwitzgebel, 2003; Schwitzgebel et al., 2006). At the same time, it is not clear that his argument targets the *transparency assumption* as defined here at all. Consider the following passage: “Although many mornings I remember a dream or two—and sometimes they seem to have been quite vivid—I can't tell you whether those dreams are in color. The historical swings in opinion about black-and-white vs. color dreaming suggest that I am not singularly inept, and that incompetence in assessing the coloration, or lack of it, of our dream life is fairly widespread, despite the considerable confidence people often exhibit when questioned on the matter. We don't know the phenomenology of dreaming nearly as well as we think we do” (Schwitzgebel, 2011, pp. 14, 15).

Here, it becomes clear that reading Schwitzgebel's skepticism about reports of dream color as threatening the *transparency assumption* rests on an equivocation between two different ways of knowing the phenomenology of dreaming, where one refers to knowledge of particular dreams, as they are remembered and reported upon awakening, and the other to general *opinions* about dreaming, reached independently of individual instances of dream recall. If his argument is intended as a critique of the transparency of individual dream reports, rather than of our general *opinions* about dreaming, then it does not follow from the available evidence. In particular, the change from reports of predominately black-and-white to predominately colored dreaming in the 1960s was accompanied by a shift from questionnaire studies to studies relying on dream reports following REM sleep awakenings (Schwitzgebel, 2011, p. 2). As Schwitzgebel himself points out, the only study to analyze the occurrence of color terms in individual dream reports from the 1940s suggests that there was no difference in *reports* of colored dreaming as compared to newer studies. Schwitzgebel concludes that “although people's *opinions* about their dreams changed dramatically, their dreams remained approximately the same” (Schwitzgebel, 2011, pp. 6f). Consequently, his position is best understood as skepticism about *opinions* about colored dreaming only, to the effect that people are prone to error when asked general questions about whether they ever, or frequently, dream in color.

Next, note that Schwitzgebel's argument for rejecting the view that dreams changed in tandem with opinions about dreaming is precisely that the analysis of individual dream reports from the relevant period suggests otherwise. Thus, his skepticism about opinions about colored dreaming is based on the *transparency assumption* for individual dream reports. More generally, this suggests that no principled version of skepticism about dream reporting can be constructed by shedding doubt on the *relative* trustworthiness of different types of reports: In order to shed principled doubt on the trustworthiness of dream reports in general, one would have to show that even under *ideal* reporting conditions, subjects systematically misdescribe their dreams. While Schwitzgebel presents convincing evidence suggesting that people's opinions about their dreams are untrustworthy, he does not—and indeed could not—show that the same is true for reports given under ideal reporting conditions.

This is in keeping with the conclusions reached by a number of researchers investigating the incidence of colored dreaming. Importantly, these studies converge on the assessment that there is a hierarchy in the trustworthiness of dream reports, with dream reports following REM sleep awakenings generally being regarded as the gold standard of dream reporting (Murzyn, 2008; Schredl et al., 2008; Hoss, 2010). Hoss notes that reports of colored dreaming are much more frequent following REM sleep awakenings in the laboratory than for spontaneously recalled dreams, concluding that “attempting to capture the actual color originating within a dream may rely on REM awakenings” (Hoss, 2010, p. 89). Similarly, Schredl et al. (2008) found that reports of black-and-white dream elements were dramatically reduced when subjects were asked to describe the color of their dreams immediately after awakening. By contrast, Murzyn (2008) failed to find a significant difference between subjects' responses to general questionnaires about dream color and their individual dream reports. Clearly, more detailed research investigating the accuracy of opinions and general assessments about dreaming (see Domhoff, 2003 for a general discussion), as well as a more careful and differentiated account of the trustworthiness of different types of dream reports is needed. Yet, progress on these questions is only possible if one endorses the *transparency assumption* for dream reports given under ideal reporting conditions: only then does one have a standard against which one can measure the *relative* trustworthiness of dream reports given under *less* than ideal reporting conditions.

My preliminary conclusion is that a more constructive reading of Schwitzgebel's argument is to say that it raises the question of when and under which conditions dream reports are trustworthy. Indeed, this interpretation is in accordance with Schwitzgebel's own acknowledgement that “despite its untrustworthiness, introspection must be given a central role in the study of consciousness. […] Behavior and physiological measures alone tell us nothing about consciousness unless it is established that those measures correlate with conscious experience; and introspection is the most straightforward way to establish such correlations” (Hurlburt and Schwitzgebel, 2007, p. 53). Understood in this way, moderate distrust about dream reports is a valuable and potentially fruitful tool for developing a positive, anti-skeptical account of dream reporting^7^.

## Why to trust dream reports

So far, we have seen that while the *transparency* and *reportability assumptions* are methodologically necessary for scientific dream research, questions about the experiential character of dreaming and the relationship between dreaming and dream reporting cannot be resolved by conceptual analysis alone. The demand for empirical evidence confirming the trustworthiness of dream reports is equally misguided. The former strategy commits the *armchair fallacy*, the latter leads to an insoluble form of skepticism. Skepticism about dream reporting also cannot be based on the disagreement between different types of reports from different subjects, because any such argument will afford *prima facie* credibility to certain types of reports^8^. Here, an important lesson is that contrasting contradictory reports can, at best, suggest which *types* of dream reports are more trustworthy than others. Hence, any non-dogmatic, anti-skeptical account of dream reporting will be cautiously and moderately skeptical.

If questions pertaining to the trustworthiness of dream reports are neither conceptual nor empirical, nor based on contradictory reports then—absent further alternatives—there is a creeping suspicion that the question of whether dream reports are trustworthy in principle might be a pseudo-problem, an artifact of a philosophical debate. Perhaps, construing the problems of dream experience and of the transparency of dream reports as genuine *questions* was misleading all along. This suggests that the questions typically raised about dreaming and dream reporting require reformulation. Rather than asking, *Are dreams experiences?*, and *Do dream reports reveal what it is like to dream (or whether it is like anything at all)?*, we should ask, *What does science reveal about dream experience?*, *and Which types of dream reports reveal what it is like to dream?*.

Anti-skepticism about dream reporting is indebted to the different variants of skepticism about dream reporting in important respects. From Schwitzgebel's argument, it inherits the insight that not all dream reports are equal in their trustworthiness. From Malcolm's analysis of dreaming and Dennett's Multiple Drafts Model, it inherits the assumption that there is indeed a deep and unbreakable link between dreaming and dream reporting, as well as the insight that in talking about dream experience, we are always only indirectly doing so, via dream reports. Yet, according to anti-skepticism, the nature of this connection is methodological, rather than conceptual or empirically investigable.

If this is correct, can anything positive by said in favor of *anti-skepticism*? Without a positive account in its support, it may seem that it is a simple act of desperation, resulting from the insight that no empirical corroboration of the trustworthiness of dream reports is possible. I want to argue, however, that a better and more satisfying account of the *transparency assumption* exists, and one that can provide it with a sound theoretical justification. In particular, I want to suggest that the *transparency assumption* is the best explanation of dream reporting. This is an initially attractive move, because inference to the best explanation (IBE) has been suggested as a powerful response to Cartesian dream skepticism (Russell, 1912/1999, p. 9; Vogel, 1990; Briesen, 2008). I claim that it provides an equally powerful response to the skeptic about dream reporting. Following Mackonis (2013, p. 977), the standard form of IBE can be reconstructed as follows:

The surprising fact, *C*, is observed.

But if *A* were true, *C* would be a matter of course.

No available competing hypothesis can explain *C* as well as *A* does.

Hence, *A* is true.

Applied to the transparency of dream reports, the initially surprising fact is the observation that people report vivid experiences upon awakening. But if these reports were transparent in the sense that they referred to experiences occurring during sleep, they would be a matter of course. The next crucial step is to show that this is a *better* explanation than alternative hypotheses (cf. Harman, 1965). In the case of dream reporting, this could be Dennett's cassette theory of dreaming, according to which dream reporting results from memory insertion, or the possibility, suggested by Schwitzgebel, that certain subject groups systematically misdescribe their dreams as colored (or as black and white) due to media influence^9^. Only if the *transparency assumption* better explains the surprising fact of dream reporting, is the inference to its truth warranted.

What, then, makes one explanation better than another? There is widespread agreement that the overall goodness (or loveliness, as Lipton puts it) of an explanation depends on its scope, precision, simplicity, and consistency with background knowledge (Lipton, 2000, 2004; Mackonis, 2013). To begin with, the *transparency assumption* is indeed simpler than the view that dream reports are the product of memory insertion, for on the latter view, one would have to claim that while waking experience reports are the result of normal memory consolidation processes, dream reports result from non-standard memory consolidation processes such as memory insertion^10^.

The received view also does a better job at explaining why dream reports describing roughly the same types of experiences as occur during wakefulness are preceded by roughly the same types of brain activity as is correlated with such experiences in wakefulness, hence allowing for a more unifying account of neuroimaging findings. Similarly, if the skeptic accepts evidence from lucid dreaming and dream-enactment behavior in RBD as showing that these dreams are experiences, his denial that the same is true for standard dreaming appears increasingly contrived and limited in scope.

The greater scope and consistency with background knowledge also allows for more precise predictions. For instance, it predicts that memory consolidation processes are state-independent, remaining the same across the sleep-wake cycle. While this is an empirical question, Marzano and colleagues argue that “the current EEG results, in suggesting that the mechanisms involved in encoding and recall of episodic memories across wakefulness and sleep are the same, undoubtedly strengthen the general notion of a continuity between waking and sleep” (Marzano et al., 2011, p. 6681). It also predicts that a cessation of dreaming should present independently of deficient memory. Indeed, there is evidence that a cessation of dreaming can present independently of amnesia (Solms, 1997), and even that amnesia patients can report sleep-onset imagery similar to that of healthy subjects (Stickgold et al., 2000). On the cassette theory, which reduces dreaming to waking memory, this seems utterly mysterious. Finally, Schwartz and Maquet (2002) have suggested that the similarity between dream bizarreness and neuropsychological syndromes associated with circumscribed brain lesions might be used to predict regional changes in brain activation during preceding REM sleep periods (e.g., reduced activation in V4 during achromatic dreams). While this has not yet been investigated, an exciting study by Horikawa et al. (2013a) suggests that by mapping dream reports onto brain activity, such predictions might indeed be confirmed (see the section “The Way Forward: Consequences for Scientific Dream Research”).

To this reasoning, one might object that I am treating precisely those arguments as supporting the *transparency assumption* that above (in the section “Daniel C. Dennett's Cassette Theory of Dreaming”), I claimed would not satisfy the skeptic. And while this is true, note that the significance of these arguments has changed along with the reinterpretation of the *transparency assumption* in the context of IBE. While these examples do not—indeed could not—present *independent* evidence corroborating dream reports, they do contribute to an overall explanatory framework that is made possible by the *transparency assumption* in the first place, thereby also increasing its overall explanatory loveliness. This central insight is nicely formulated by Putnam: “assuming that dreams take place ‘in physical time’—i.e., that they start and stop at some time or other—various things *become* inductive evidence that correlations hold: correlations between the things we do with our eyes, muscles, vocal cords, as we sleep *and* the dream events; and correlations between the neural processes that normally go with “seeing” certain things and dream events” (Putnam, 1986, p. 317). Making certain default assumptions about dreaming and dream reporting, then, is not at odds with scientific dream research—it is the very condition for investigating dreams in a naturalistic framework. Within an IBE framework, this strategy is justified, in part, by its success. As Froese and colleagues note, “one effective way to evaluate the scientific validity of the phenomenological results of first- and second-person methods is to see whether they help us to do better science or not” (Froese et al., 2011, p. 39).

An important consequence is that the explanatory goodness of a given explanation is a revocable property. It is always possible that a better explanation might come along (van Fraassen, 1980) or that an alternative explanation is better supported by future findings. Consequently, a prediction for future research is that because the *transparency assumption* makes new forms of evidence about dreaming and dream reporting available, its explanatory goodness should further improve over time.

## The way forward: consequences for scientific dream research

Anti-skepticism about dreaming and dream reporting aims to put skeptical worries about the trustworthiness of dream reports to rest and to provide theoretical justification for the methods employed in scientific dream research. Its contribution to scientific dream research, consequently, is purely theoretical. It does suggest, however, a particular reading for report-based studies of dreaming. In particular, it frees scientific dream research from providing an answer to what can now be seen to be a misconstrued question: dream research cannot, and need not, provide a positive answer to the philosophical question of whether dreams are experiences, nor need it rule out skeptical hypotheses such as Dennett's cassette theory (cf. Kramer, 2007)^11^. Skepticism about dream reporting is a philosophical problem; it is not, however, exclusively a philosopher's problem. In particular, dream researchers, by portraying their reliance on dream reports as a weakness and proposing different ways of corroborating or even moving beyond dream reports, occasionally make dream research appear more vulnerable than it is, thus inadvertently perpetuating skeptical doubts. It is true that scientific dream research cannot *prove* dream reports to be trustworthy; but this is neither due to a shortcoming of particular research findings, nor to the poor trustworthiness of dream reports, but to the fact that the demand for empirical corroboration itself is misguided.

A more productive question for scientific dream research, and one on which much progress has been made, is under which circumstances dream reports should ideally be gathered. Importantly, the *transparency assumption* should not be construed as lending undifferentiated support to the transparency of all dream reports, but is restricted to dream reports gathered under ideal reporting conditions. What exactly these conditions are is an important empirical question. Many factors influence differences in dream recall, such as setting, method and timing of awakening, interpersonal situation, the precise wording of questions asked, etc. (Hall and Van de Castle, 1966; Winget and Kramer, 1979; Domhoff, 1996, 2003; Kramer, 2007), and their investigation will likely place further constraints on the conditions under which dream reports should be elicited. For present purposes, the main lesson is that this question is wholly separate from philosophical problems pertaining to the principled trustworthiness of dream reports.

Yet, some general remarks are possible. First, there is widespread agreement that ideal reporting conditions depend on a principle of temporal proximity: the smaller the temporal lag between dreaming and dream reporting, the more trustworthy the report. This is why laboratory awakenings are taken to be the gold standard of dream reporting (e.g., Schredl, 2010).

Second, different types of reports can be fruitfully compared to each other in order to determine their *relative* trustworthiness. On this view, transparency is not an all-or-nothing affair; rather, the *relative* transparency of different types of dream reports is determined by their ability to *approximate* that of dream reports gathered *under ideal reporting conditions*. This is important because in most studies, reporting conditions are, in fact, less than ideal: laboratory studies of dreaming are too costly and time-consuming to be widely available, and the laboratory situation itself may have a disturbing effect on dreams (Schredl, 2010).

Third, determining ideal reporting conditions may be closely related to the level of *detail* and *specificity* required by a given study. Returning to the debate on dream color, Schredl et al. (2008) found that the percentage of reports describing black-and-white dreaming not only dropped for dreams reported immediately upon awakening, but also when subjects were presented with the option that they may have forgotten dream color. Relatedly, Murzyn (2008) found that between 10 and 20% of subjects reported mixed dreams involving *both* colored and black-and-white elements. Because this result is so unexpected, neither forgetting, nor wake-state bias, nor media influence seem to be likely explanations. Generally, asking subjects to report *specific* aspects of their dreams, for instance by using affirmative probes or questionnaires about individual dreams, rather than asking them to give detailed free dream reports, can change theoretical thinking about dreaming considerably (cf. Nielsen, 2010): Merritt et al. (1994) found that the frequency of reported dream emotions increased 10-fold when subjects were asked to report their emotions on a line-by-line basis compared to free dream reports, suggesting that the prevalence of emotion was underestimated in many earlier studies. Generally, requiring too much detail might decrease motivation or lead subjects to forget or underreport those aspects that researchers are really interested in. For instance, asking subjects to keep a simple checklist log of their dreams may be the best method for assessing spontaneous dream recall frequency (Zadra and Robert, 2012), but would be wholly inadequate for investigating subjects' dreams in any detail.

Aside from detail and specificity, dream reports also differ as to their *expressive granularity*^12^. For instance, a subject might give a detailed and specific verbal description of visual dream imagery, but still be unable to express certain aspects of the experience. Here, the expressive granularity of reports can be increased by asking subjects to produce dream drawings or compare their dreams to photographs with different degrees of color saturation, brightness, and clarity (Rechtschaffen and Buchignani, 1992).

Very generally, one might say that an important goal for scientific dream research consists not only in determining the relative transparency of different types of reports and improving reporting conditions, but also, ultimately, in using dreams reported under (approximately) ideal reporting conditions to minimize the gap between reportable and actually reported dreams. Because the *reportability assumption* says that dream research is methodologically constrained by the space of reportable dreams and that any claims about unreportable experiences are unscientific, improving reporting conditions is an important strategy for mapping and analyzing the space of reportable dream experience as systematically as possible.

Another interesting question is how scientific dream research can profit from the broader debate on first-person reports. A particularly promising perspective, and one that might counteract researcher-induced bias, might be for researchers to collaborate with the participants of a given study when developing the categories or questions that later structure their dream reports. This method has been successfully used in experimental neurophenomenology (Lutz et al., 2002; Thompson, 2007). A similar strategy was used in a self-observation study of hypnagogic imagery, where different categories of sleep-onset experiences were correlated with neuromuscular events and EEG measures (Nielsen, 1991–1992, 1995). Sleep onset may be a particularly promising target for mapping fine-grained EEG stage scoring to fine-grained experiential categories (Hori et al., 1994).

A related question is whether the trustworthiness of dream reports can be improved through training. Cultivating a disciplined approach to experience, as in certain contemplative traditions, may stabilize attention to ongoing experience and render first-person reports more accurate (Varela, 1996; Overgaard et al., 2008). Lucid control dreams might be a particularly promising candidate. Yet, the reliability of different induction techniques is variable (Stumbrys et al., 2012) and it is unclear whether the results from lucid dreaming can be generalized to nonlucid dreams (Voss et al., 2013). In nonlucid dreams, the attenuation of reflective thought, memory and control over attention and volition (Hobson et al., 2000) makes ongoing introspection impossible. So while subjects can certainly be trained to be better dream *reporters*, it is not clear that training has any direct and controllable effect on dreaming, at least not without changing the target phenomenon. On the plus side, this ensures that dream experience is largely undisturbed as it unfolds and thus protected from many of the pitfalls of ongoing introspection in wakefulness (Lyons, 1986)^13^. In this respect, dream reports are better compared to paradigms using immediate retrospection, such as Hurlburt's descriptive experience sampling (cf. Hurlburt and Schwitzgebel, 2007 for a critical discussion).

Another particularly intriguing area of research, and an important step toward the integrated investigation of dreaming and waking experience, is the comparison of reports across the sleep-wake cycle. This method has already been used to investigate the frequency of thinking and hallucinations across the sleep-wake cycle (Fosse et al., 2001) and could also be applied to the comparative study of dreaming and waking imagination or mind wandering (Wamsley, 2013; Fox et al., 2013). But it could also be used to investigate, in a systematic and controlled manner, whether dream recall really is the fleeting, unstable phenomenon it has often been described as. A possible outcome could be that dream reporting is, in fact, no less trustworthy than reporting waking experience. Rechtschaffen, one of the pioneers of dream research, suggests as much, claiming that “When laboratory subjects are awakened from the REM stage of sleep, they generally have little difficulty in giving a fairly long, detailed report of dreams, with transcripts sometimes running to several typed pages. Indeed, it is often easier to get detailed, articulate reports of ASCs [altered states of consciousness] than detailed reports of normal waking consciousness” (Rechtschaffen, 1975, p. 143). At least two studies suggest that this observation can be confirmed in controlled studies. Stickgold et al. (2001) collected a total of 1748 reports from 16 subjects over a period of 14 days. During the daytime, subjects responded to a pager, at night, their sleep stages were monitored by a nightcap sleep monitoring system. Aside from between-subject variation, they found that median report lengths varied more than 2-fold over the sleep-wake cycle, with REM reports being the longest, followed by reports from active wakefulness, quiet wakefulness, NREM sleep, and sleep onset. This finding has recently been replicated by Siclari et al. (2013). In addition, when they asked their subjects how far back in time they could recall the content of experience and how rich and complex it was (measured by how long it would take to recount it), they found the same pattern, with the highest rating occurring for REM sleep reports. If such findings were further substantiated in the future, might this suggest that dreaming, far from being a particularly clear target for skepticism about first-person reports, is in fact more readily recalled than waking experience? Whether or not this is the case, engaging in such comparative studies of dream and waking experience reports only makes sense on the *transparency assumption*.

Finally, it has long been suggested that given the uncertainties inherent in dream reporting, progress might ultimately be made by avoiding the study of dream reports altogether. In 1966, Hall and Van de Castle wrote that “conceivably, advances in instrumentation will eventually permit dreams to be ‘televized’ as they are being dreamed. If this time ever comes, we will no longer have to ask people to tell us their dreams because we can record and study the dream exactly as it was experienced” (Hall and Van de Castle, 1966, pp. 18–19). A similar idea underlies Revonsuo's (2006, pp. 300–303) science fictional thought experiment involving the so-called dream-catcher test, in which he envisions researchers constructing a 3D model of a subject's dreams based exclusively on measures of brain activity and comparing it to a 3D model based exclusively on data gathered from dream reports (for a similar proposal, see Perogamvros, 2013).

**Figure 2:**
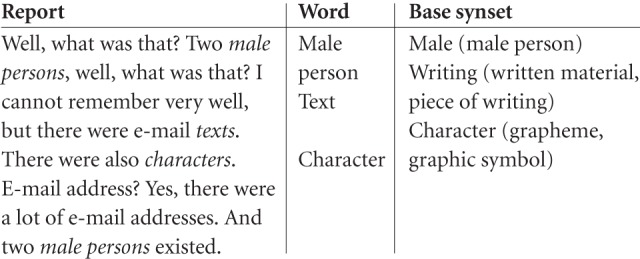
**Example of a dream report used in the construction of a “dream-reading machine” (Horikawa et al., 2013b, p. 25)**.

Again, a recent study seems to suggest that this is not pure science fiction. Horikawa et al. (2013a) gathered approximately 200 reports from sleep onset from 3 subjects sleeping in an fMRI scanner. Words describing visual scenes or objects were extracted from the reports and mapped to a lexical database. Then, internet photos corresponding to the visual object categories (or synsets; see Figure 2) were shown to the participants while they lay awake in the scanner, in order to train a computer program to associate the object categories to specific patterns of activity in the visual cortex. Based on a further round of sleeping in the scanner and dream reporting, the researchers then used this program to predict with around 60% accuracy the visual objects described in the dream reports^14^.

Does this suggest that researchers might indeed move *beyond* dream reports in the future? This is not the place to engage in science fictional predictions as to whether “dream reading” independently of dream reporting might someday be scientific practice. I merely want to point out that in developing such technologies, dream reports play an indispensable role both during training sessions and to establish the accuracy of the researchers' predictions. At present, predictions only count as successful if they are confirmed by dream reports. Far from corroborating the trustworthiness of dream reports, dream reports, under the assumption of transparency, corroborate the accuracy of the predictions and thus are instrumental in developing “dream-reading” technologies in the first place. It seems, then, that at least in this respect, the methodological background assumptions of scientific dream research have not changed since the discovery of REM sleep in the 1950s—though they are certainly being put to innovative and exciting uses.

Another point, however, may be even more important. Above, I noted that anti-skepticism about dream reporting predicts that as dream research progresses, the explanation it provides of dream reporting should further improve. Horikawa et al. (2013a, p. 640) note that because they were able to successfully predict visual dream experiences from cortical activity patterns observed during visual stimulus presentation in wakefulness, their findings support a “*principle of perceptual equivalence*,” according to which perception and dream imagery share a common neural substrate. Because only one previous study involving signal-verified lucid dreams investigated the brain activity underlying specific dream contents (Dresler et al., 2011), the importance of this finding is hard to overestimate. Assuming that they accurately predicted not just dream reports, but visual dream experience, this would be an important step toward enriching an account of which types of experiences occur in dreams, and of how to relate dream experience to standard wake states (Windt and Noreika, 2011). By the same token, it would also increase the loveliness of anti-skepticism and its ability to provide a unified explanation of reports of dream and waking experience.

## Conclusions

In this article, I used different variants of philosophical skepticism about dream reporting to defend a positive account of the trustworthiness of dream reports. According to my anti-skeptical account, dream reports, when gathered under ideal reporting conditions, are trustworthy with respect to conscious experience during sleep. The *transparency assumption* initially has the status of a methodologically necessary default assumption, but is theoretically justified because it offers the best explanation of dream reporting. Seen in this way, it is the condition of possibility for scientific dream research. While this is a decidedly anti-skeptical view, it inherits important insights from the discussed variants of skepticism about dream reporting, such as the recognition of a close, unbreakable connection between dreaming and dream reporting and the view that not all dream reports are equal in their trustworthiness. While the questions of whether dream reports are trustworthy in principle and whether dreams are experiences are shown to be misguided, many more specific and empirically investigable questions concerning the ideal conditions of dream reporting or the question of which experiences occur in dreams can now be formulated, suggesting important perspectives for future research and for the comparative study of dreaming and waking experience.

I want to end on a more speculative note. A recurrent theme of this paper has been that skepticism about dream reporting can be regarded as a proxy war on more general issues. Skepticism about dream reporting is typically raised in the context of more general variants of skepticism about first-person reports, with dream reports being regarded as a particularly clear and vulnerable target for skeptical attacks. Because all of the discussed variants of skepticism about dream reporting generalize to skepticism about first-person reports, and because they all fail with respect to dreaming, related worries about first-person reports in general appear equally ungrounded. At the very least, it seems plausible to assume that the case for skepticism about first-person reports cannot be made in any of the ways discussed here. Consequently, if the philosophical debate on dream reporting can indeed be regarded as a test case for skepticism about first-person reports, then it might be promising to assume that my positive, anti-skeptical account of dream reporting generalizes to first-person reports of waking experience as well. If so, then perhaps scientific dream research might be better described as a particularly clear example of the importance of trusting first-person reports—if only because its central reliance on first-person reports is perhaps even more obvious than for other branches of consciousness research. At the very least, this hopefully suggests that an integrated approach to first-person reports across the sleep-wake cycle is a promising and important goal for future research.

### Conflict of interest statement

The author declares that the research was conducted in the absence of any commercial or financial relationships that could be construed as a potential conflict of interest.

## Footnotes

^1^In laboratory studies, ~85% of REM sleep awakenings result in dream recall (Nielsen, 2000), but most subjects only occasionally remember their dreams at home, suggesting that only 5–10% of dreams are spontaneously recalled (Domhoff, 1996. p. 41).

^2^An early formulation of this idea is suggested in (Windt, 2010); a more detailed discussion can be found in Windt (in press).

^3^Epistemic transparency is intended in a non-technical, metaphorical sense; in particular, it should not be confused with the thesis that the mind is transparent to itself (see Carruthers, 2011 for discussion). Transparency is a visual metaphor, intending to capture the intuition that dream reports are the closest researchers can come to “watching the sleeping mind” (Cartwright, 2010, p. 17). Transparency is also meant to reflect the fact that dream reports are not identical with, but better conceived of as separate from dreaming. Finally, transparency reflects the historical situation that the theoretical problems raised by dream reporting were nearly invisible throughout most of the history of philosophical theorizing about dreaming.

^4^Note that most contemporary dream researchers accept that dreams, at least occasionally, occur during all stages of sleep (Nielsen, 2000). Because the deep stages of NREM sleep, in particular, lack the wake-like EEG activity that characterizes REM sleep, the claim that dreams occur in these stages of sleep is exclusively based on dream reports. In fact, it has been suggested that contrasting reports of dreaming and nondreaming from NREM sleep may be a particularly fruitful method for identifying the correlates of dreaming within, rather than across, sleep stages (Noreika et al., 2009).

^5^The *armchair fallacy* is related to Dennett's *Philosophers' Syndrome*, or the practice of “mistaking a failure of imagination for an insight into necessity” (Dennett, 1991, p. 401). While both result from exaggerated confidence in apparently necessary conceptual truths, the *armchair fallacy* does not result from a failure in exercising imaginative abilities, but rather from a disregard for the phenomenal character of experience, coupled with a tendency to derive conclusions about phenomenal experience from conceptual analysis alone.

^6^Indeed, Squires (1973) argues that the *experiential assumption* should be rejected because it cannot be straightforwardly confirmed by empirical evidence.

^7^In Windt (in press) I argue that a similarly constructive reading can be applied to Rosen's (2013) narrative fabrication thesis of dreaming.

^8^One might object that if researchers found contradictory evidence, for instance of colored dreaming, among reports elicited following REM sleep awakenings, the situation would be different: in this case, this disagreement would support principled skepticism about dream reporting. However, this objection fails to acknowledge the possibility of genuine variability, e.g., in colored and black-and-white dreaming, both between different subjects and across different dreams. As Hohwy (2011) has argued against Schwitzgebel, variability in experience reports provides no grounds for distrusting reports, because this reported variability is equally well, or perhaps even better, explained by variability in experience.

^9^ Because the cassette theory poses the more radical threat to the received view, and because Schwitzgebel's skepticism has already been restricted to opinions about colored dreaming, I focus on the former example here.

^10^Recall, however, that Dennett takes the opposite to be true, because he claims that the received view requires a presentation process in addition to a composition and a memory-loading process. I do not think, however, that proponents of the received view need accept the claim that ongoing dream experience is wholly separate from dream composition: perhaps, the dreamer's ongoing expectations and emotional reactions shape the unfolding dream narrative. Lucid control dreams would seem to support this view. See Windt (in press) for a detailed discussion.

^11^The same applies to older theories claiming that dreams occur instantaneously at the moment of awakening, such as the Goblot theory; (see Kramer, 2007, pp. 17–31 for discussion).

^12^I borrow this term from Sascha Fink.

^13^A related question is whether the efficiency of studies can be improved by using subjects with high dream recall. Given the apparently poor association between dream recall frequency and personality traits, there seems to be a case for using high recallers (Domhoff, 2003); however, it is also possible that different people have different types of dreams, and Ruby et al. (2013) recently found evidence for neurophysiological trait differences between high and low recallers.

^14^The reconstruction of dynamic visual images based on imaging data from waking brain activity obtained during the presentation of YouTube movies was first achieved by (Nishimoto et al., 2011).
